# Multiomics integration reveals NETosis heterogeneity and TLR2 as a prognostic biomarker in pancreatic cancer

**DOI:** 10.1038/s41698-024-00586-x

**Published:** 2024-05-20

**Authors:** Yifan Fu, Jinxin Tao, Yani Gu, Yueze Liu, Jiangdong Qiu, Dan Su, Ruobing Wang, Wenhao Luo, Tao Liu, Feifan Zhang, Taiping Zhang, Yupei Zhao

**Affiliations:** 1 General Surgery Department, State Key Laboratory of Complex Severe and Rare Diseases, Peking Union Medical College Hospital, Chinese Academy of Medical Sciences and Peking Union Medical College, Beijing, 100730 China; 2 4 + 4 Medical Doctor Program, Chinese Academy of Medical Sciences and Peking Union Medical College, Beijing, 100730 China; 3 State Key Laboratory of Common Mechanism Research for Major Diseases, Department of Biochemistry and Molecular Biology, Institute of Basic Medical Sciences Chinese Academy of Medical Sciences, School of Basic Medicine Peking, Union Medical College, Beijing, 100005 China; 4 Department of Computer Science, University College London, London, UK; 5 Clinical Immunology Center, Chinese Academy of Medical Sciences and Peking Union Medical College, Beijing, 100730 China

**Keywords:** Cancer microenvironment, Prognostic markers

## Abstract

Pancreatic ductal adenocarcinoma (PDAC) is a highly malignant neoplasm characterized by a poor prognosis and limited therapeutic strategy. The PDAC tumor microenvironment presents a complex heterogeneity, where neutrophils emerge as the predominant constituents of the innate immune cell population. Leveraging the power of single-cell RNA-seq, spatial RNA-seq, and multi-omics approaches, we included both published datasets and our in-house patient cohorts, elucidating the inherent heterogeneity in the formation of neutrophil extracellular traps (NETs) and revealed the correlation between NETs and immune suppression. Meanwhile, we constructed a multi-omics prognostic model that suggested the patients exhibiting downregulated expression of NETs may have an unfavorable outcome. We also confirmed *TLR2* as a potent prognosis factor and patients with low *TLR2* expression had more effective T cells and an overall survival extension for 6 months. Targeting *TLR2* might be a promising strategy to reverse immunosuppression and control tumor progression for an improved prognosis.

## Introduction

Pancreatic ductal adenocarcinoma (PDAC) represents a principal contributor to cancer-associated mortality, with a mere 10% of patients demonstrating survival at a five-year interval post-diagnosis^1^. Primary surgical resection is feasible in less than 20% of patients, while the majority manifest with advanced, non-resectable disease^2,3^. Intriguingly, even among patients deemed suitable for surgical intervention and subjected to neoadjuvant therapy, an estimated 75% will encounter recurrence within a biennial timeframe, with a near 20% five-year overall survival rate^4,5^. The implementation of immune therapeutic strategies has been largely futile due to the immunologically “cold” tumor microenvironment (TME) characterized by significant myeloid cell infiltration and impeded T cell activation^6^.

Neutrophils, as a dominant constituent of myeloid cells and one of the most abundant immune effector cells of the human immune system, garnered increasing amounts of interest over recent years^7–10^. These cells demonstrate considerable plasticity, possessing the ability to adapt to shifts within the tumor immune microenvironment (TIME) via mechanisms such as phagocytosis, degranulation, and the formation of neutrophil extracellular traps (NETs), thereby establishing a reciprocal regulation^9–11^. Diverse stimuli can induce the release of NETs via a unique cell death process known as NETosis, which is distinct from both necrosis and apoptosis yet closely linked to autophagy^9,12,13^. Unlike most types of tumors, pancreatic cancer necessitates autophagy for tumor growth, and the inhibition of autophagy leads to an increase in reactive oxygen species (ROS) stress and mitochondrial oxidative phosphorylation, which subsequently results in significant tumor regression and improved survival outcomes^14^. Various stimuli mediated by ROS can regulate multiple cytotoxic mechanisms in neutrophils, such as NETosis, autophagy, and ferroptosis^15–17^. These mechanisms exerted by neutrophils are significant contributors to the immunosuppressive phenomena observed within the TIME, subsequently impacting the prognosis of patients with pancreatic cancer. Simultaneously, in the TIME of pancreatic cancer, the interactions among neutrophils, macrophages, and T cells also play a significant role in immune suppression. However, the research on this mechanism, particularly the immune suppression induced by neutrophils, remains unclear and necessitates further investigation. Previous studies have demonstrated the importance of neutrophil infiltration in the PDAC TME, however, most of them were incapable of inspecting neutrophils at single cell level due to high sensitivity and vulnerability when sampling and sequencing^18^. Moreover, neutrophil subtypes were not clearly identified except for specific pro-inflammatory phenotypes, and only a few targets including *IL17*, *TIMP1* were characterized to illuminate the relationship between NETosis and PDAC considering a lot of genes were advanced involving NETosis^18–21^.

**Fig. 1:**
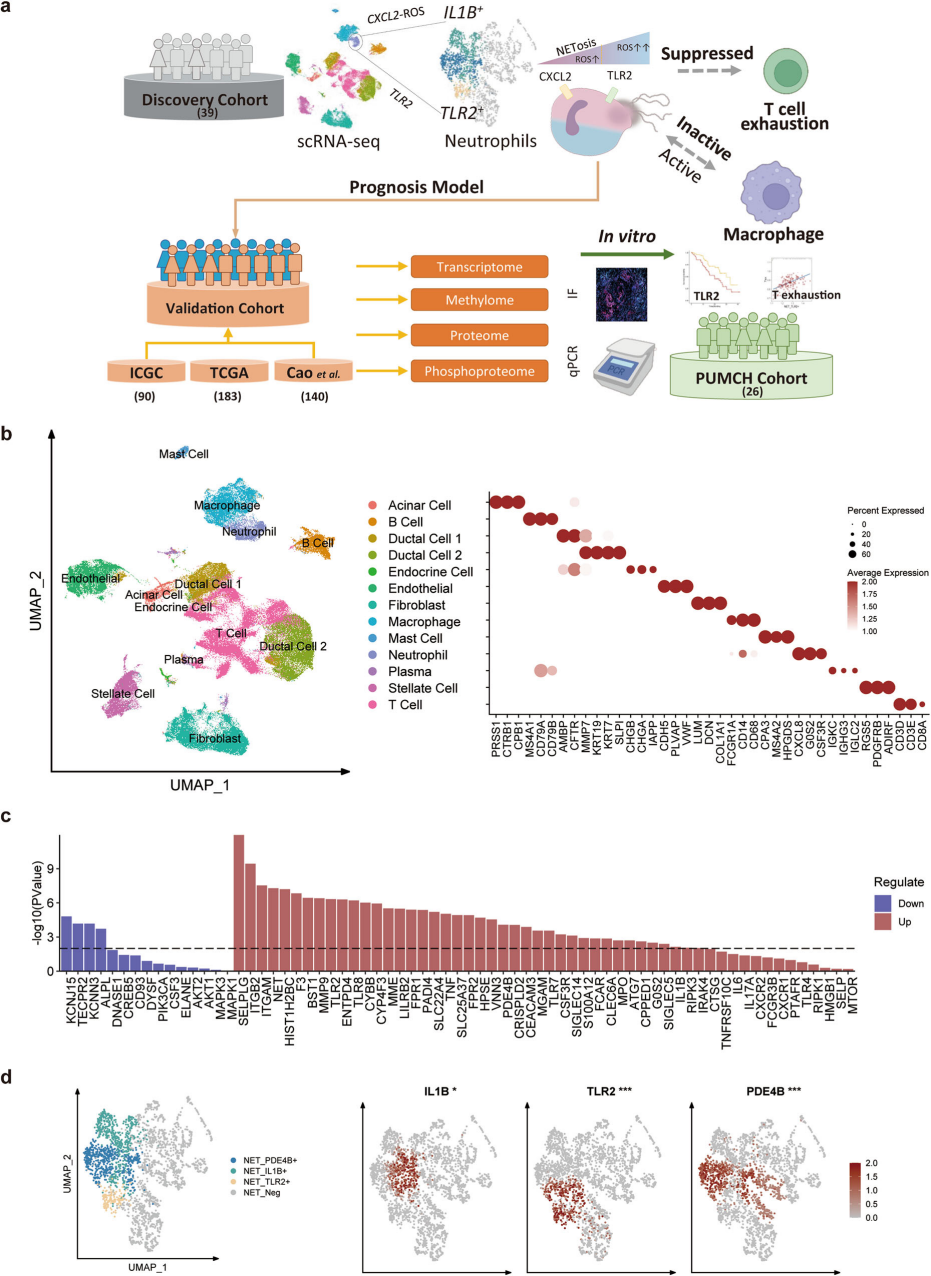
Single-cell transcriptomic analysis in PDAC discovery cohort. **a** Overall design and workflow of experiment in this study; **b** UMAP reduced plot characterizing the integrated cell map, consisting of 13 annotated cell types together with representative marker genes; **c** The regulation of genes of NET signature in tumor samples. The dotted dashed line indicated *p* = 0.01; **d** NMF reduction of neutrophils. Three subtypes defined by NET signature were highlighted and the expression of their representative genes (*IL1B*, *PDE4B*, and *TLR2*) was shown on the right side.

Here, we retrieved from published literature and in-house patients, generating three cohorts to explore the heterogeneity of NETosis in the PDAC microenvironment. These cohorts were used to validate the clinical relevance of newly discovered NET subtypes and to integrate multi-omics datasets, respectively (Fig. 1a). We conducted a comparative analysis of the heterogeneity between different subtypes of NET positive and NET negative neutrophils in terms of reprogramming metabolism, tumor clearance, and immune suppression. We particularly focused on the heterogeneity of TIME suppression by NETosis activation, and how they are influenced by ROS levels. Moreover, we constructed three distinct NET subtype signatures based on their expression profiles and built a prognostic model, which was validated across all cohorts. Additionally, we identified individual genes from the signatures as potential robust prognostic targets, demonstrating equivalent prognostic power in the PDAC, and confirmed the association of key targets with clinical prognosis and immune suppression in our in-house cohort through immunofluorescence (IF) staining and quantitative real-time polymerase chain reaction (RT-qPCR) verification.

## Results

### Single-cell RNA seq characterizes the heterogeneity of NETosis in PDAC Neutrophils

Based on the cohort described in the method section, the first discovery cohort was utilized to comprehensively catalog the populations of each cell type. After pre-processing and correcting the batch effect, 103, 116 cells passed quality control and were used for the downstream analysis (Supplementary Fig. 1a). A total of 13 cell types were identified according to well-known cell type marker genes from previous studies^22,23^ (Fig. 1b).

To inspect the heterogeneity of NETosis in PDAC neutrophils, a neutrophil gene signature consisting of 69 genes was generated and divided into two parts: neutrophil activation and NETosis. Given that NETs underlie the pro-cancer effects within most cancers, we first asked whether NET signature can distinguish tumor samples from healthy control in PDAC. The NET signature enrichment score was calculated in the tumor and healthy group and showed an obvious difference, which was in accordance with existing results^21^. However, when we explored the tendency inner the NET signature, we surprisingly found most genes had the same regulated trends with the NET signature, however some single genes were inversely downregulated in tumor groups (Fig. 1c). It suggested the potential of distinct functions or heterogeneity during NETosis.

To retrieve neutrophils from the primary dataset, a total of 2, 624 tumor neutrophils were extracted from 39 patients to further investigate. After correcting the batch effect, these cells were confirmed to be distributed across all 39 patients with reasonable heterogeneity from each dataset (Supplementary Fig. 1b). NMF and unsupervised clustering algorithm were utilized to identify 7 sub populations of neutrophils based on the NET signature and take the most significantly different genes as the representative of NET positive subtypes (*PDE4B*, *IL1B* and *TLR2*), while the others were defined as NET negative neutrophils (Fig. 1d, Supplementary Fig. 1c and 1d). In addition, the NET score was calculated in the tumor and control group, and the tumor group exhibited a higher NET score (Supplementary Fig. 1e). To identify the expression profile of these NET positive subtypes, three signatures were generated and can similarly distinguish the tumor from the control group (Fig. 1d). Wang et al. ^18^ represented a criterion that clustered neutrophils, and we aligned our NET-based subtypes with their signatures (Supplementary Fig. 1f). We found NET_PDE4B+ neutrophils were similar to a combination of TAN-2 and TAN-3 from Wang et al., while other neutrophils didn’t exhibit paired expression patterns, which might be affected by neutrophil capture process and tumor heterogeneity.

### The activation of Neutrophil exhibits metabolic and gene reprogramming specificity

**Fig. 2:**
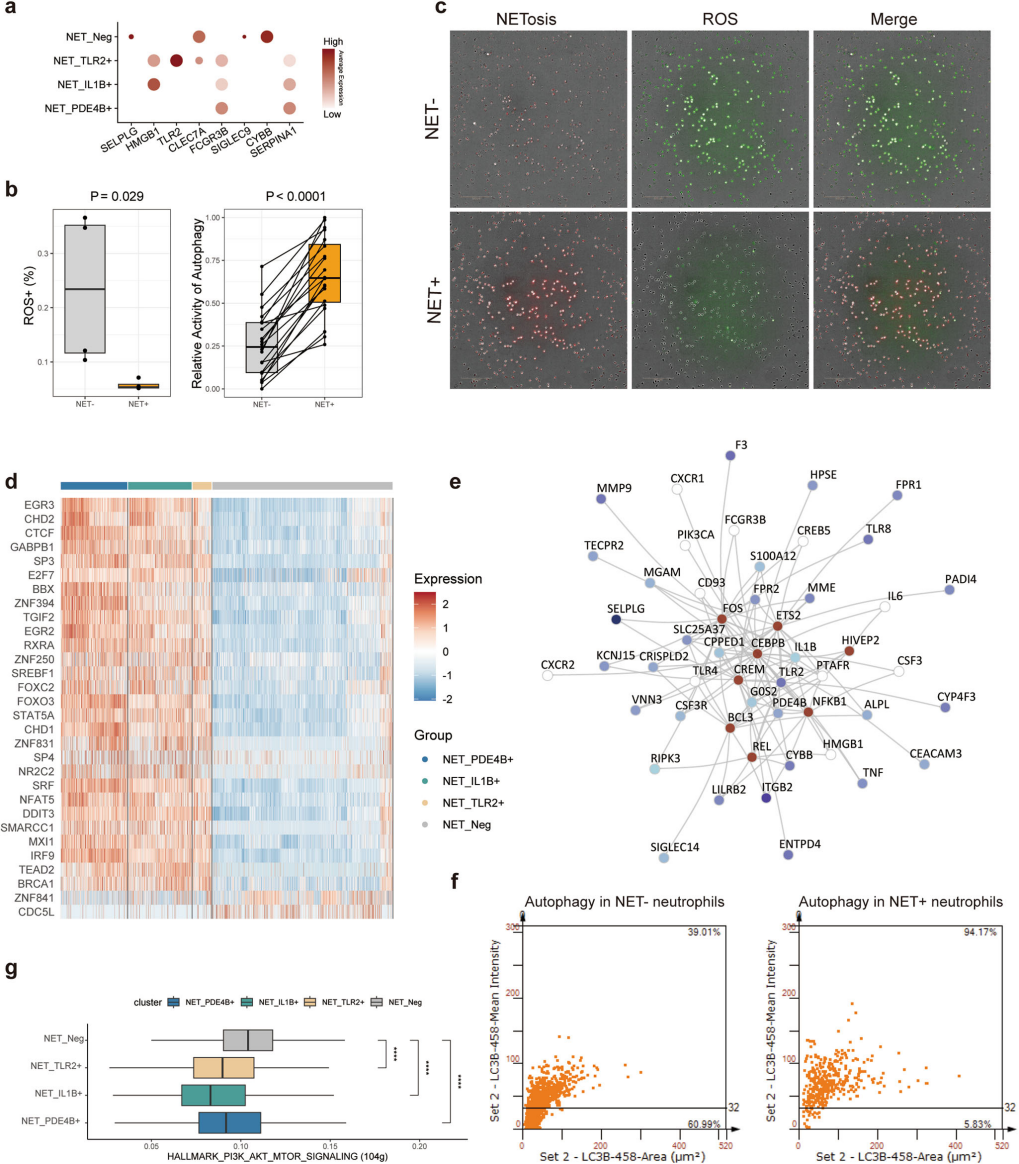
Characterization of gene and metabolic reprogramming of different neutrophil subtypes. **a** Dot plot exhibits the relative expression of the representative NETosis activation genes and key metabolic genes; **b** Evaluation of ROS level and the activity of autophagy in PDAC patients; **c** Measuring the ROS level in NET positive and negative neutrophils, red color for ROS level and green for NETosis; **d** TFs regulatory activity of different neutrophil subtypes; **e** NETosis related gene regulatory network reveals the vital co-regulated TFs (marked by dark red) and their target NETosis related genes (marked by the *p*-value of different between the tumor group and normal group); **f** The scatter plot shows the percentage of LC3B^+^ MPO^+^ neutrophils (NET positive neutrophils in autophagy) and LC3B^+^ MPO^-^ neutrophils (NET negative neutrophils in autophagy) in PDAC tumor tissues; **g** PI3K-AKT-mTOR pathway activity observed higher in NET_Neg neutrophils.

Copious cell surface receptors have been shown to activate NETosis^9^. To explore whether the heterogeneity of NET positive subtypes was formed by distinct activation pathways inducing NETosis, we observed the relative expression of different pathway activated receptors, involving *SELPLG* (P-selectin), *HMGB1* (platelets), *TLR2* (bacteria), *FCGR3B* (immune complex). Simultaneously, several pertinent genes related to NETosis were measured including *CLEC7A* (ductin-1), and *CYBB* (NOX2) (Fig. 2a). Besides the upregulated of *CYBB* in NET_Neg neutrophils, it also showed significantly different expression patterns among different NET positive subtypes, suggesting heterogenous NETosis induced conditions.

Of note, NADPH oxidase gene *CYBB*, a critical origin of ROS, was detected upregulated in NET negative neutrophils (Fig. 2a). Relative lower ROS stress with a significant difference was observed in NET positive subtypes. In consideration of the ROS stress induced switch of autophagy^24^ and ferroptosis^17^, we next asked whether there ROS stress difference between different subtypes of neutrophils. We found the ROS pathway activation of NET negative neutrophils was significantly higher than other NET positive subtypes and we also observed higher ROS levels in NET negative PDAC patients (Figs. 2b, 2c and Supplementary Fig. 2a).

To explore the potential regulated mechanism, transcript factors (TF) analysis was executed and revealed the different regulation target genes between NET positive neutrophils and NET_Neg neutrophils. Neutrophils in the NET positive subtype exhibited higher regulatory activity in inflammatory and cell proliferation pathways, including *EGR3*, *CHD2*, *TGIF2*, *FOXO3*, *STAT5A* and *NFAT5* (Fig. 2d). It suggested an intrinsic regulatory difference between NET positive and NET negative neutrophils. We also wondered whether representative genes were vital connective in the gene regulatory network, and their co-regulated TFs were selected to construct the NETosis related gene regulatory network (Fig. 2e). We confirmed that most NETosis genes with the ability to distinct tumor and normal groups were jointly regulated by several TFs including *REL*, *NFKB1*, *FOS*, *BCL3*, *CREM*, *CEBPB*, *HIVEP2* and *ETS2*. It is worth mentioning that representative genes were located very central to the NETosis related gene regulatory network, which provided confidence to take these genes to downstream analysis. Meanwhile, we found the activity of hypoxia, glycolysis and oxidative phosphorylation was upregulated, which indicated a ROS-related metabolic reprogramming in NET positive neutrophils (Supplementary Fig. 2b, 2d).

Next, the higher ROS score in NET_Neg neutrophil suggests the high-level ROS would lead to inhibition of autophagy rather than NETosis. Through IF staining and live-cell ROS and NETosis fluorescence detection, we found significantly downregulated autophagy in NET negative neutrophils, which had higher ROS levels than NET positive neutrophils (Fig. 2b, c, f).

To explain the potential regulatory mechanism of autophagy, we evaluated the activity of the mTOR pathway, which is widely reported to inhibit autophagy^25,26^. The high activity of mTOR provided evidence for the inhibition of autophagy in NET_Neg neutrophils (Fig. 2g, Supplementary Fig. 2c). At the same time, we also found the upregulated of TNF-α via NF-κB and inflammatory response pathway in NET positive neutrophils, suggesting the different anti-tumor mechanism caused by NETosis (Supplementary Fig. 2b).

### NET induced immune suppressed by inactive interaction with macrophage and T cell exhaustion

**Fig. 3:**
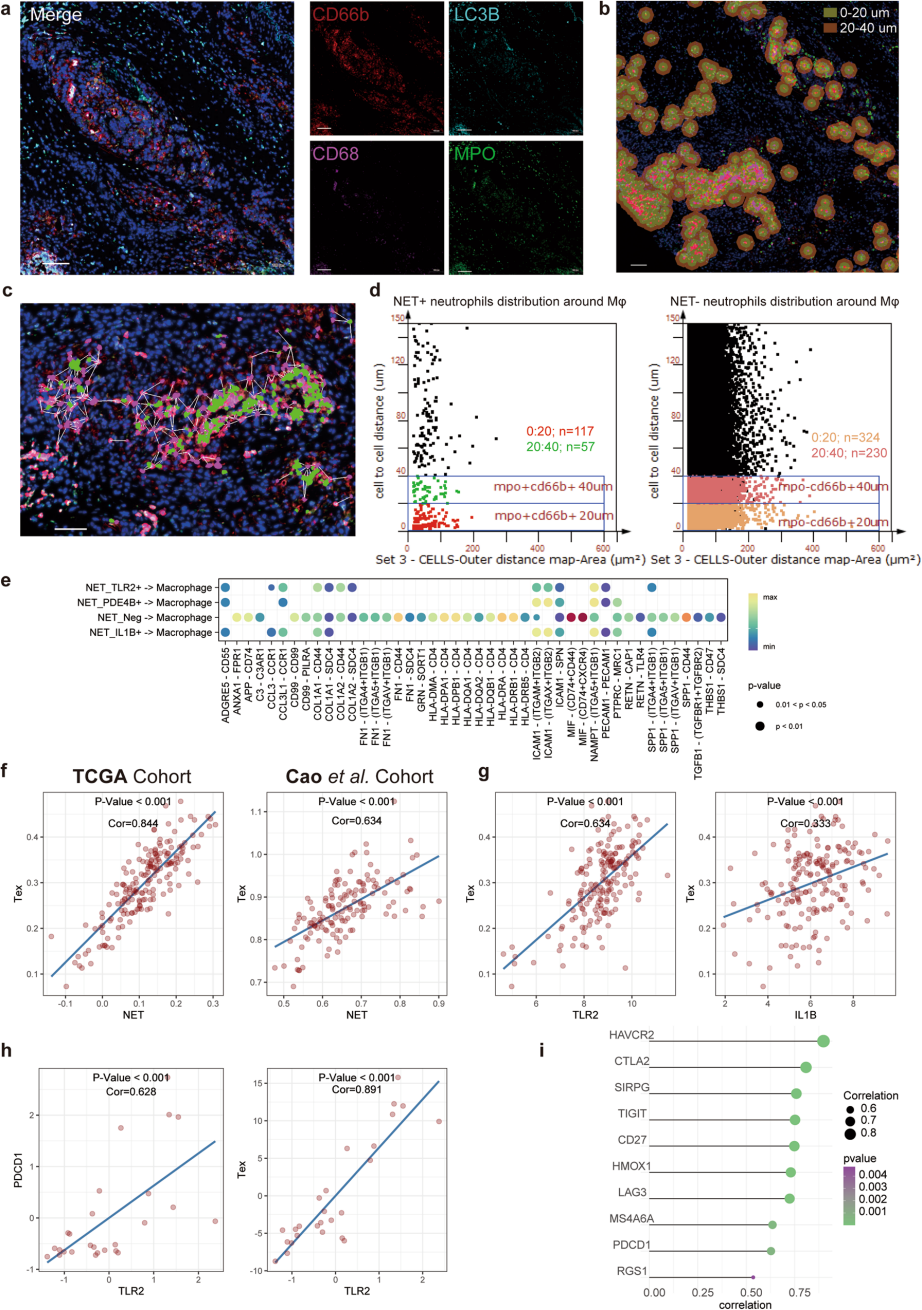
Heterogeneities of TME including neutrophil-macrophage interaction and neutrophil induced T cell exhaustion. **a** Representative multi-color staining showing CD66b (red, neutrophil marker), CD68 (purple, macrophage marker), LC3B (light blue, autophagy marker), MPO (green, NETosis marker), and DAPI (blue, nucleic acid dye) in PDAC tumor tissues, scale bar = 100 µm; **b** StrataQuest software recognizes macrophages in tumor tissue by CD68 staining. Quantify the number of neutrophils at different distances from macrophages: 0-20 μm (dark green area), 20-40 μm (brown area), scale bar = 100 µm; **c** StrataQuest software was employed to quantify the positional relationship between NET negative/positive neutrophils and macrophages, scale bar = 50 µm; **d** The counts of NET positive/negative neutrophils in 20 μm and 20-40 μm area from macrophages; **e** Dot plot profiled ligand and receptor from neutrophils to macrophages, and the color of the dots showed the connection strength; **f** Linear model profiles the correlation between NET score and T cell exhaustion score. The left panel showed results from the TCGA-PAAD cohort, while the right one showed the cohort from Cao et al.; **g** The Linear model between *TLR2* and *IL1B* with T cell exhaustion score in TCGA-PAAD cohort; **h** and **i** Validation in PUMCH cohort patients by qPCR and profiling of *TLR2* with other T cell exhaustion markers.

Given that immune suppression was partly induced by remodeling of TME^27^, we further investigate the heterogeneity of interaction between NET subtypes and macrophage. The interaction between macrophage and NET negative neutrophils was significantly active compared with NET positive subtypes (Supplementary Fig. 3a). To provide more evidence for the interaction between neutrophils and macrophages, we applied IF staining in PDAC tissues, and identified the distance between NET positive neutrophils/ NET negative neutrophils and macrophages (Fig. 3a, b). The distance was indicated and calculated to count the neutrophils within 20um and 40um from CD68^+^ macrophages (Fig. 3b, c). It was confirmed that macrophages were surrounded by a greater number of NET negative neutrophils than NET positive neutrophils (Fig. 3d, Supplementary Fig. 3b), and it’s consistent with the result from transcriptome data.

To discover which signals predominately cause the inactive in NET positive subtypes and ask whether there was heterogeneity between different positive subtypes, we visualized outgoing and incoming signal pathways (Supplementary Fig. 3c). Compared to NET negative neutrophils, NET positive neutrophils were downregulated in several antigen-processing pathways such as MIF, CCL and MHC-II, while the relative strength of IL1, ICAM, CCL, CXCL and PECAM1 were inversely upregulated, (Fig. 3e, Supplementary Fig. 3c and 3d).

Moreover, we surprisingly found the expression pattern of NET_PDE4B+ neutrophils and NET_IL1B+ neutrophils were similar, so it was reasonable to investigate whether there existed a potential development sequence of NET positive neutrophils (Fig. 3e). Hence, we constructed pseudotrajectory and excitingly found the two lineages of NET (Supplementary Fig. 3e). The pseudotrajectory showed the first lineage generated from NET negative to NET_TLR2+, and another lineage was from NET negative to NET_PDE4B+ and terminally ended with NET_IL1B+. Meanwhile, we examined the correlation among *IL1B*, *TLR2*, and *PDE4B* expression patterns. Consistent with our hypothesis, only *IL1B* and *PDE4B* showed significant correlation while others didn’t (Supplementary Fig. 3f), which provided confidence to define the isogeneity of NET_IL1B+ and NET_PDE4B+ neutrophils.

Moreover, it is also widely acknowledged that the dysfunction of T cells in PDAC TME contributed to immune suppression. So, the subsequent T cell activation was investigated independently. The correlation between NET signature scores, marker gene expression and T cell exhaustion scores was tested with the linear model in the validation cohorts (Fig. 3f). The NET score showed a significantly positive correlation with T cell terminal exhaust, suggesting a T cell dysfunction immune suppressed microenvironment.

To further validate the correlation between NETosis with T cell exhaustion, we investigated the correlation between T cell exhaustion and NET signatures in vitro. The expression of *IL1B* and *TLR2* was inspected with T cell exhaustion score in the same linear models. The correlation between *TLR2* and T cell exhaustion score was significantly higher than *IL1B* (Fig. 3h). Hence, *TLR2* was selected to observe potential immunosuppression in clinical patients. All tumor samples from the in-house cohort were included, where the expression levels of *TLR2* and genes related to T cell exhaustion were measured. It was found that *TLR2* is significantly positively correlated with almost all genes associated with T cell exhaustion as well as T cell exhaustion score (Fig. 3h, i).

### Multi-omics prognostic model based on NET signature

**Fig. 4:**
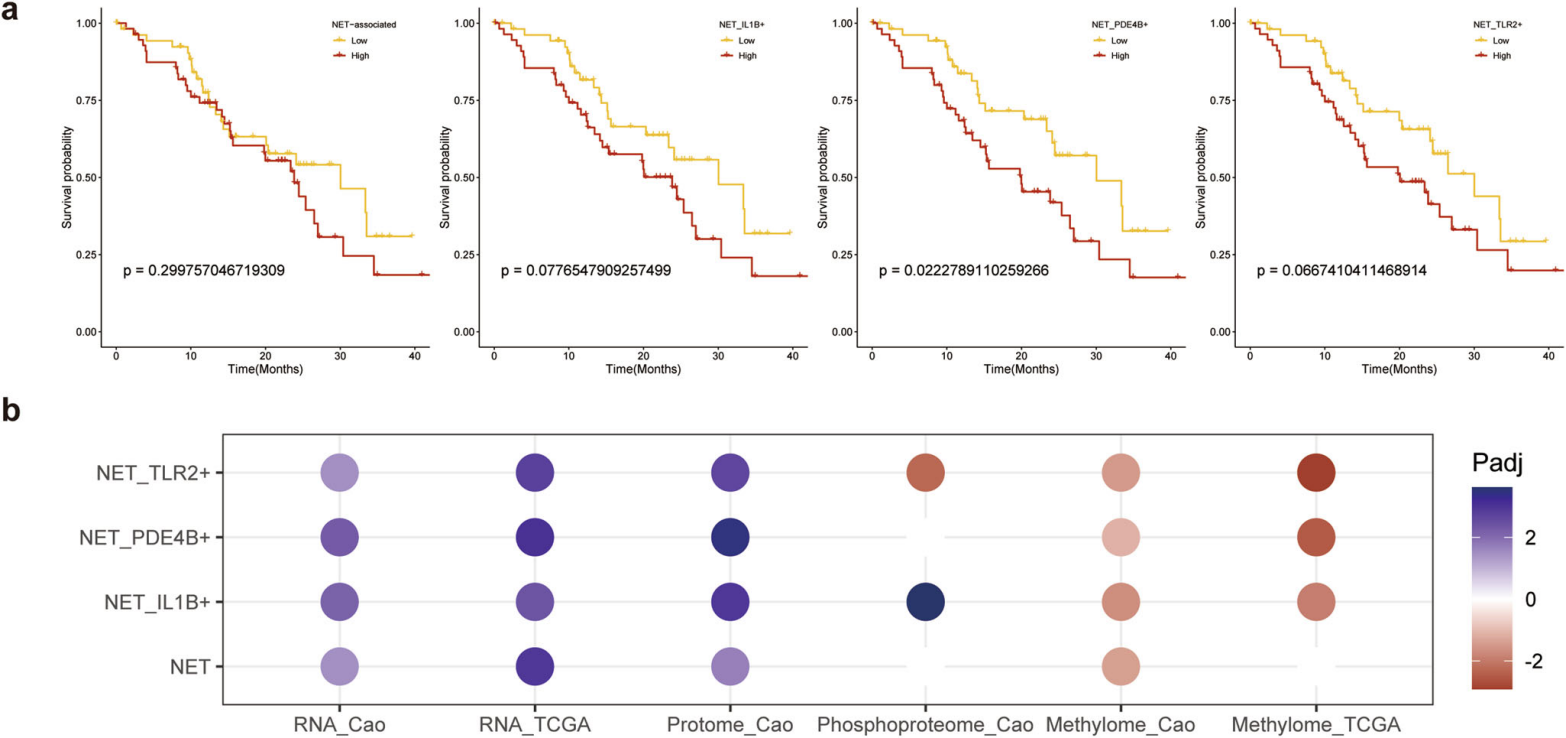
Evaluation of NET signature-based prognosis model. **a** K-M survival analysis reveals the potential of NET signatures. NET_IL1B+, NET_TLR2+, NET_PDE4B+ score high group had more favorable outcomings, while NET score cannot tell the difference in survival; **b** Multi-omics including transcriptome, proteome, phosphoproteome and methylome data were integrated to prognosis the survival time of patients. The color showed whether the pattern indicated favorable (red) or unfavorable (blue) outcomes. Only adjusted *P*-value less than 0.01 data was shown.

To further analyze whether NET signatures were associated with clinical outcomes in PDAC, validation cohorts were included to test the power of prognosis. First, the RNA-seq expression matrix of the cohort from Cao et al. was inspected. Thrillingly, the NET signature cannot act as an efficient prognosis indicator, while the separated NET signatures of NET_PDE4B+ were determined as independent indicators for overall survival and the NET_IL1B+, NET_TLR2+ signatures showed a potential trend despite not reaching the threshold (*p* = 0.078) (Fig. 4a). The same result was observed in the TCGA-PAAD cohort that the NET score is not an efficacy prognosis feature with *P*-Value = 0.245. It provided evidence that the whole NET signature, or all NET related genes, might not exerted a consistent effect to predict clinical outcomes.

Further multi-omics integration data from validation cohorts were used to confirm the power of NET signatures in an even larger scope. Transcriptome, methylome, proteome and phosphoproteome datasets were retrieved with clinical data. To fit large-scale dataset, the grouping criteria were reset to autofit with default parameters. Interestingly, we verified the effect power of NET subtype signatures and confirmed the inefficient prognosis power of the NET signature in most datasets (Fig. 4b). It was worth noting that the association between phosphoproteome and clinical outcomes showed no significance in the NET signature, while a contrary efficient result between NET_TLR2+ and NET_IL1B+, which indicated the intrinsically heterogenous influence of NETs on the prognosis.

### *TLR2* had equivalent prognostic power to NET signatures

Considering the advantage and convenience of inspecting single gene rather than multi-gene signatures in clinical application, we further wondered whether single gene from signatures had the potential power to predict clinical outcomes after constructing the prognosis models with several NET signatures. Given that three representative genes of NET signatures were the most upregulated NETosis gene in each cluster from NMF reduction and significantly made a distinction between tumor and normal group, they were chiefly taken into consideration.

Before investigating clinical outcomes, the specificity of expression in neutrophils was observed (Supplementary Fig. 4a). It was observed that the expression of *IL1B* and *TLR2* was specific to neutrophils while *PDE4B* was poor in specificity and also not efficient in prognosing the outcome, so *PDE4B* was dropped in the downstream analysis (Supplementary Fig. 4b).

**Fig. 5:**
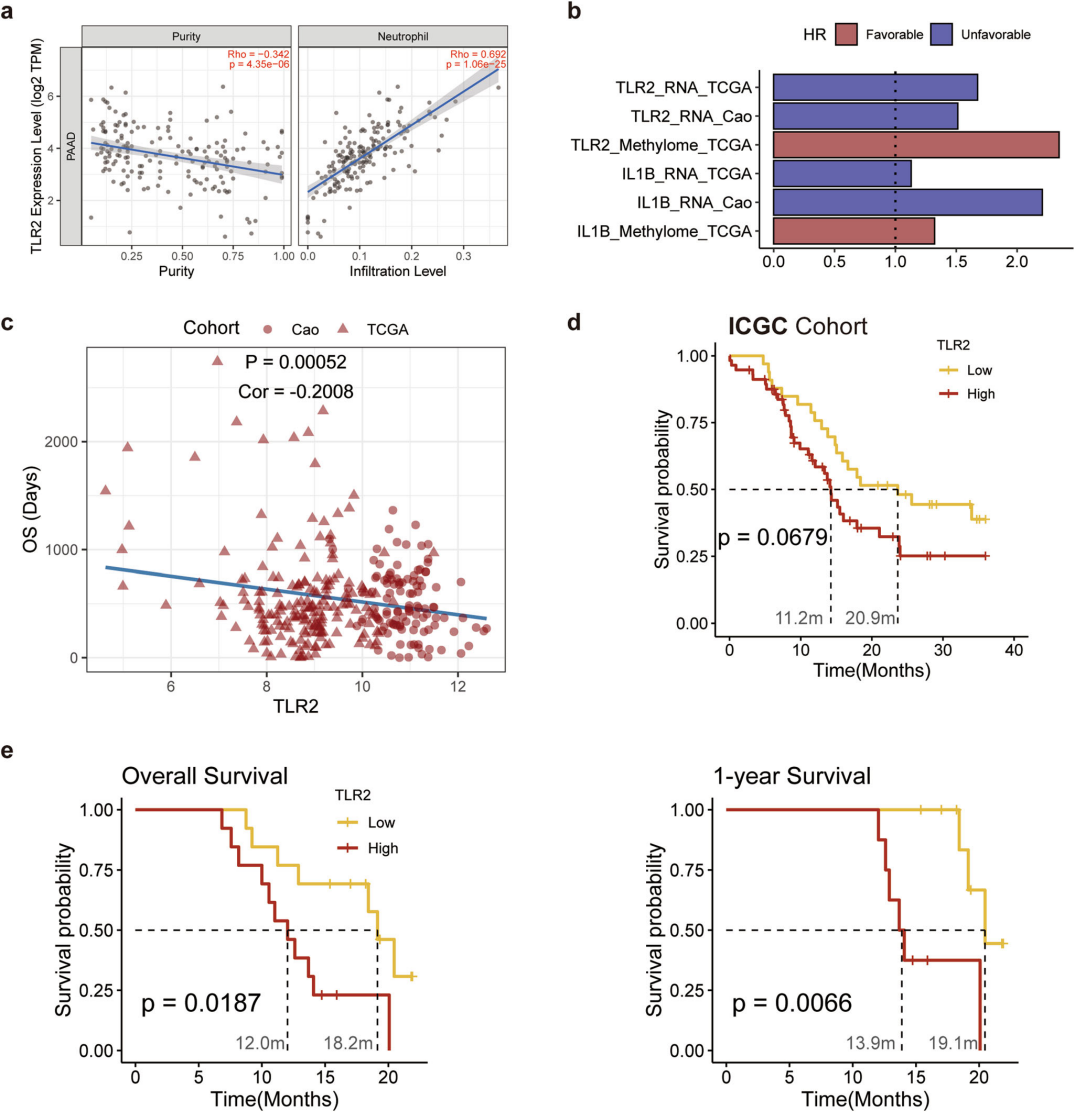
Evaluating the effectiveness and accuracy of the single gene prediction model. **a** Immune infiltration analysis; **b** Selected single gene as prognosis feature. The color showed whether the single gene was related to favorable (red) or unfavorable (blue) clinical outcomes. TCGA and Cao et al. validation cohorts were included; **c** A linear model was established by *TLR2* expression and overall survival, which suggested upregulated in *TLR2* neutrophils lead to unfavorable clinical outcomes; **d** K-M survival analysis validation in ICGC cohort; **e** The expression of *TLR2* in the inhouse cohort exhibited power to predict the overall survival and 1-year survival.

Meanwhile, considering a few macrophages also expressed these genes, to further determine whether the single gene can equivalently characterize the expression status of neutrophils, we explored the correlation between the expression of single gene, the infiltration level of neutrophils, macrophages and purity of the tumor in TCGA cohort. Similar to the analysis of single-cell, *IL1B* and *TLR2* expression were significantly associated with purity and neutrophil infiltration, while not correlated with infiltration of macrophages (Fig. 5a, Supplementary Fig. 4c). Furthermore, spatial RNA sequencing results also revealed adjunct rather than overlap between *IL1B*^+^, *TLR2*^+^ spots and macrophage-enriched spots marked by *CD68*^+^ (Supplementary Fig. 4d).

All the above results indicated that *IL1B* and *TLR2* were promising equivalent prognostic factors in PDAC. Hence, we can investigate single gene expression of *IL1B* and *TLR2* in bulk sequencing results to infer the expression pattern in NET_IL1B+ and NET_TLR2+ neutrophils.

To validate the prediction effectiveness, single genes were used as independent prognosis factors. In excellent agreement with the specificity of *IL1B* and *TLR2*, single genes showed efficient prognostic power in validation cohorts, and *PDE4B* was alike inefficient in predicting the outcomes (Fig. 5b). Considering the location of these proteins, TLR2 is a membrane receptor protein, while IL-1β (encoded by *IL1B*) is a secretory protein, which was poor in specificity in proteome validation. We also confirmed that IL1B protein abundance was ineffective in distinguishing clinical outcomes (Fig. 5b). Hence, we turned to TLR2 in multi-omics cohorts.

Consistent with transcriptome results, the expression of *TLR2* was linearly correlated with OS in validation cohorts (Fig. 5c), which suggested the *TLR2* expression was a promising target to predict the clinical outcome at the early stage of PDAC. We also observed the prognosis power of *TLR2* in methylome cohorts and a highly methylated TLR2 was associated with a favorable outcome (Fig. 5b).

Next, we would like to evaluate whether the single gene of *TLR2* can predict the clinical outcome in the real world. The ICGA cohort was utilized to inspect the correlation between the expression of TLR2 and the overall survival of patients recorded in Australia. Patients with higher expression of TLR2 were found to survive 9 months shorter than another group (median OS 11.2 months vs. 20.9 months), which meant the OS was reduced by nearly half (Fig. 5d). We next collected the in-house cohort samples and divided them into TLR2^high^ and TLR2^low^ groups. Consistent with our hypothesis, the overall survival time and 1-year survival time were significantly 6 months longer in the TLR2^high^ group (Fig. 5e).

## Discussion

TME in PDAC is both fascinating and challenging, composed of numerous populations of fibroblasts, dense extracellular matrix, dysfunctional vascular system and heterogenous suppressive immune cells^3^. Moreover, targeted and immune-based therapies show very limited prospects since PDACs are largely resistant to these agents and have lower response rates compared to other cancers^2,28^. A major reason is that in the TME, T cells are devoid and exhibit low activation and refractoriness to checkpoint blockade, aggravating the adaptive immune^6,29,30^. Nevertheless, abundant neutrophils provide a promising target of immunotherapy and have been substantiated the efficiency of tumor regression and extend survival, considering a prominent myeloid cell infiltration in TME^18,31–33^.

Despite of the substantial neutrophil infiltration in PDAC, previous studies scarcely illuminated the presence of neutrophils in the single cell RNA-seq results. Such bias leads to unclear characterization of neutrophils in PDAC although bulk RNA-seq revealed the association between neutrophil infiltration and unfavorable prognosis. Moreover, due to the short lifetime of neutrophils, it also remains detection to clarify the subtypes of neutrophils.

Here, we utilized the single-cell RNA sequence, spatial RNA sequencing combined with multi-omics data, revealing the potential heterogeneous induction and cytotoxicity mechanisms of NETosis, which indicated different clinical outcomes in several comprehensive cohorts.

Several studies had demonstrated the key role of neutrophils in the TME, but the diverse functions including pro/anti-tumor, pro/anti-metastatic were ambiguous and immune context depended^34^. In PDAC, neutrophil infiltration was associated with unfavorable prognosis and was hypothesized to promote tumor growth and metastasis resulting in a vicious circle^15,18^. It has been widely reported that NETosis is a key pathway to exert the functions in neutrophils^35^. Many stimuli can activate neutrophil NETosis via different mechanisms such as immune complex, bacteria and platelets^9^. Recently, several studies targeted NETosis manifested promising results to reverse tumor growth as well as improve immunotherapy^20,36^. To inspect whether NET is effective in predicting the clinical outcomes in PDAC, we extracted the 2, 624 tumor neutrophils from the dataset and a signature of 69 NET-associated genes was subsequently constructed with neutrophil activation and NETosis genes (Figs. 1b, 1c and 1d). Compared with healthy patients, the NET score in tumor patients was significantly higher in accord with the distinct functions of NETosis in the TME (Supplementary Fig. 1e). Interestingly, when comparing the single genes from the signature between tumor and health samples, genes displayed an inversed tendency (Fig. 1c). Upregulated of integrin, selectin and matrix metalloproteinases while downregulated of ion channel, tectonin, suggested the inner heterogeneity of NETs in PDAC neutrophils. Combined with previous distinct activation of NETosis, we thereby hypothesize that neutrophil infiltration in PDAC TME can be activated by several PDCA TME specific stimuli via different mechanisms and have a heterogenous impact on clinical outcomes.

NMF was applied to dims reduction and three outstanding marker genes from the NET signature were selected, including *PDE4B*, *IL1B* and *TLR2*. Tracing back to the NET signature, *IL1B* and *TLR2* were from the NETosis subset, while *PDE4B* was from the neutrophil activation subset. Pancreatic cancer is one of the leading cachexia tumors, approximately 70 percent of PC patients have cachexia and progress to unfavorable outcomes^37^. Interleukin-1β (IL1B) secreted from neutrophils was reported in pancreatic cancer and was found related to cancer cachexia by induing upregulated expression of *LCN2* in the *Lcn2-KO* mouse model^38^. Phosphodiesterase 4B (PDE4B) was reported as regulated by *BHLHE40* which was a direct regulator of the pro-tumor gene and played a key role in the polarization of neutrophils in PDAC^18^. Toll-like receptor 2 (TLR2) can regulate the immune environment, and was considered a promising target therapeutic drug-delivery pathway for its pattern recognition function and had been studied in several tumors^39,40^. Besides, intestinal microbes could migrate to the pancreas through the pancreatic duct and induce immune response by toll-like receptors^41^, which might also contribute to the occurrence of PDAC.

Of note, a recent study by Wang et al. proposed a criterion that divided PDAC TME neutrophils into several types according to the glucose metabolic states and provided outstanding gene markers of each subtype^18^. To begin with, we applied these genes and invested the expression pattern in our dataset (Supplementary Fig. 1f). As mentioned before, neutrophils had relatively low sequencing depth due to their rapid self-digestion. According to the expression pattern, we matched our NET_PDE4B+ neutrophils with TAN-2 and TAN-3, NET_IL1B+ neutrophils potential with TAN-3. However, NET_TLR2+ and NET_Neg neutrophils unlikely matched TAN-1 to TAN-2 from the previous study. Regarding NET_Neg, or TAN-1 like, neutrophils exhibited the resemble active glycolysis metabolic pathway and hypoxia feature (Supplementary Fig. 1f, Supplementary Fig. 2d), confirming the similarity of such population. The unmatched neutrophils suggested the neutrophils in the PDAC TME were instinct of heterogeneity and drove us to explore the function and potential of the other neutrophil population.

Hereafter, we examined the change of NETosis and its effect on the PDAC TME. NET_Neg neutrophils were found to express active glycolytic, and oxidative phosphorylation pathways, which contributed to high metabolic activity in response to the higher ROS stress (Fig. 2b, c and Supplementary Fig. 2d). As comparable, the other neutrophils express a moderate ROS stress, which may contribute to NETosis and eventually transfer to distinct function and TME. Recent studies have widely reported this contradictory standpoint that ROS had both anti-tumor and pro-tumor effects^12,24^. Chan et al. confirmed that NETosis was ROS-dependent in the pancreatic adenocarcinoma mouse model^42^. We observed the higher ROS and lower activity of autophagy in the NET negative neutrophils (Fig. 2b, c and 2f). Hence, we concluded that under moderate ROS stress, neutrophils are activated to form NETs in response to immune stimuli in the microenvironment. In contrast, under high ROS stress conditions, the internal environment of neutrophils becomes highly unstable, and the upregulated mTOR signaling might inhibit autophagy while activating other mechanisms of cell death such as ferroptosis^17,25,26^. However, the precise mechanism should be further evaluated.

Suppressed immune cell communication is another pivotal reason for immune desert PDAC TME^43,44^. NET_Neg neutrophils were found higher activity to communicate with macrophage, while this communication between NET positive neutrophils and macrophage was suppressed (Supplementary Fig. 3a, 3c). Of note, *CXCR2* was found highly expressed in NET_IL1B+ and NET_PDE4B+ neutrophils, which was reported as a tumor *CXCL1* target gene and also can induce T cell terminated (Supplementary Fig. 3c)^45^. At the same time, a recent study also observed the expression of *CXCL2* can induce the migration of neutrophils from the peripheral circuit to TME and might account for the liver metastasis after gemcitabine treatment, which might form feedforward recruitment of anti-tumor neutrophils and melatonin was validated to induced the expression of tumor cells rather than macrophages to recruit the neutrophils by upregulated *CXCL2*^42,46^. The following observation provided sufficient evidence for the key immune regulation role and the intrinsic heterogeneity of NETosis.

We further suspect whether NET_IL1B+ and NET_PDE4B+ have a potential development relationship and confirmed by gene expression, pseudotrajectory and defined NET_TLR2+ neutrophils as a distinct terminal of NETosis (Supplementary Fig. 3e, 3f). According to the previous study, NET_PDE4B + , or TAN-2 like, neutrophils located in the middle stage of neutrophil development without distinctive functional features^18^. Moreover, we observed the wide expression of PDE4B in our scRNA-seq dataset (Fig. 4a), thereafter we took IL1B and TLR2 and their NET signatures as vital consideration in the subsequent construction of prognosis models. Other studies characterized NETosis as suicidal NETosis and vital NETosis, which were involved by CXCR2-ROS and TLR2 respectively^47^. We observed the similar expression of CXCR2 as well as relatively activated NF-κB pathway in NET_IL1B+ and NET_PDE4B+ neutrophils while the lack of expression in NET_TLR2+ subtype, which provided more confidence for us to set our neutrophil NETosis criterion and our basic hypothesis (Supplementary Fig. 2b, 2c).

Although neutrophil infiltration has increasingly been invested to perform PDAC prognosis, it’s still controversial whether NETosis leads to favorable outcomes or not. We also confirmed the inapparent survival of all NET signatures if not considering NET subtypes (Fig. 4a). Here, we based on comprehensive neutrophil subtypes, divided according to the NETosis, and several NETosis signatures constructed a prognosis model with several multi-omics datasets. We confirmed the unfavorable overall survival of high NET_IL1B+ , NET_TLR2+ signature scores (Fig. 4a). Meanwhile, transcriptome, methylome and proteome datasets exhibited almost similar prognostic efficacy in all NET signatures. However, the phosphoproteome was reciprocal in NET_IL1B+ and NET_TLR2+ signatures, which corresponded with our hypothesis that different NETosis had intrinsic heterogeneity on clinical outcomes.

Taking advantage of the specificity of *IL1B* and *TLR2* in the neutrophils (Supplementary Fig. 4a), we further discovered whether the single-gene prognosis model had equilibrium efficacy of prognosis. The expression of *TLR2*, *IL1B* exhibited similar and powerful prognosis ability in transcriptome. However, as a secretory protein, IL-1β was poor in specificity to link to neutrophils, while TLR2, a membrane protein, was suitable for validation in proteome data. Another methylome cohort exhibited a tight correlation between methylation of *TLR2* and overall survival (Fig. 5b), which was consistent with the finding that abnormal methylated would influence the activation of neutrophils and lead to diseases^48,49^.

Moreover, *TLR2* was demonstrated to participate in the activation and regulation of CD8^+^ T cell by serving as a co-stimulatory molecule in mTOR signaling and might ultimately regulate CD8^+^ T cell exhaustion under the stress of immunotherapy^50,51^. The expression of *TLR2* was found tightly correlated with T cell terminal exhaustion and clinical outcomes. Further validation in in-house patients exhibited 6 months longer survival expectation in TLR2^high^ patients, which extended the survival period by nearly 50%.

Overall, we distinguished neutrophils by the criterion of NETosis and observed the intrinsic heterogeneity of different NETosis subtypes as well as the dependency of appropriate ROS stress to induce NETosis. Moreover, we characterized the immune suppression caused by inactive communication between NET positive neutrophils with macrophages and their contribution to T cell exhaustion. Ultimately, we constructed a prognosis model to evaluate the clinical outcomes based on NET signatures and provided abundant evidence for the heterogenous neutrophil subtypes divided by NETosis.

However, several limitations exist. Due to the extremely short lifetime of neutrophils, we don’t validate the immune suppression mechanism in vivo, which will be explored in our further exploration. Also, all patients in this work were primary PDAC should be taken into consideration before reaching the ultimateness.

## Methods

### Cohort selection

In house clinical specimens were collected from September 2020 to September 2022, a total of 26 freshly frozen surgically resected pancreatic cancer specimens with prognostic information were collected from patients without preoperative treatment at the Peking Union Medical College Hospital (PUMCH). The diagnoses were confirmed by histopathology. The overall survival data were obtained through electronic medical records or telephone follow-up.

Furthermore, three independent cohorts with PDAC from published literature were used to carry out integrative analysis. All the individuals were treated naïve at the time of sequencing for the primary tumor. The first cohort consisted of single-cell transcriptomics data from CRA001160^22^, GSE154778^52^ and GSE212966^23^, downloaded from TISCH^53^ and NCBI Gene Expression Omnibus (GEO) platform (https://www.ncbi.nlm.nih.gov/geo/) respectively, which was used for discovery purpose. The spatial RNA sequencing data was also downloaded from GEO platform. The second cohort was extracted from The Cancer Genome Atlas (TCGA) and the gene expression data, DNA methylation data and corresponding clinical information of the TCGA-PAAD cohort were downloaded from UCSC Xena^54^. The third cohort included gene expression, proteome, phosphoproteome and methylation was downloaded from Cao et al. together with clinical information^55^. The International Cancer Genome Consortium (ICGC) cohort (PACA-CA project, 138 patients) clinical information and RNA expression data were downloaded from the ICGC data portal. The latter three cohorts were included to validate, or act as valid cohorts.

### Ethics statement

This study was approved by the Institutional Ethics Committee of PUMCH (Ethic code: I-22PJ487) in accordance with the ethical guidelines of the Institutional Ethics Committee and with the 1964 Declaration of Helsinki and its later amendments or comparable ethical standards. Written informed consent forms were signed and obtained from all participants.

### RNA extraction and quantitative real-time polymerase chain reaction (RT-qPCR) analysis

Total RNA was extracted from 26 freshly frozen pancreatic cancer specimens from in house cohort by Trizol reagent (Invitrogen, 15596018). The cDNA was synthesized by PrimeScriptTM RT Master Mix (TaKaRa, RR036A) and the quantitative PCR was conducted by the TB Green Fast qPCR Mix (TaKaRa, RR430S) according to the manufacturer’s protocol. The primers used for quantitative PCR are summarized in Supplementary Table 1. Each experiment was conducted three times. The mRNA expression of target genes was calculated using the 2-Δct method relative to the housekeeping gene GAPDH. Then, the expression level was normalized by using the scale function in R^56^.

### NET signature construction

During NETosis, numerous key genes are proposed involving diverse responses. Here we referred Zhang et al. construction of the NET signature with two parts, neutrophil activation and NETosis^21^. In brief, a total of 69 genes were converged as the NET signature to estimate the NETosis and identify the subtype heterogeneity of neutrophils in terms of NET (Supplemental Table 2).

### Single-cell RNA-seq data processing

Raw expression matrix from each dataset was imported into R software to filter out low-quality cells ( > 10% mitochondria genes, <200 genes/cell or >5000 genes/cell) and merged using the ‘Seurat’ R package (v4.3.0). Three adjacent samples from GSE212966 were excluded for low quality. Next, gene expression levels were normalized and scaled to all genes. A total of 2, 000 highly variable genes were used to conduct PCA reduction dimension. To correct the batch effect, the Harmony R package was used with the top 20 PCs. For primary analyses, unsupervised cell clusters were acquired by graph-based clustering approach (The top 20 dims were selected, resolution = 1.0), and visualized by UMAP dimensionality reduction. The clusters were subsequently annotated according to the expression of canonical markers in previous literature^22,23^. Marker genes of each cluster were identified by the FindAllMarkers function under the criteria of log fold change larger than 0.5.

### Batch effect correction

All data were merged and the batch effect was corrected. First, the ScaleData function (Seurat package) was used to eliminate potential batch effects caused by gene expression. Then the Harmony algorithm (RunHarmony function in harmony package V0.1.1) was further applied to correct batch effects for each dataset, which was a widely used, sensitive and accurate integration of single-cell data algorithm^57^. Finally, when the neutrophils were subset from the whole dataset, we used the SCTransform function (Seurat package) to eliminate batch effects based on the labels of the datasets. The cell arrangement was visualized before and after the batch effect correction to demonstrate no clear batch effect.

### Non-negative matrix factorization

Non-negative Matrix Factorization (NMF) was performed using the NMF R package (v0.26) to identify underlying expression programs of NETs. Neutrophils from tumor samples and genes from NET signature with batch correction were subset to NMF procedure and independent of patient selection. For neutrophils, the NMF reduction was used for downstream dimensionality reduction and visualization with all dims and resolution of 0.5 for fewer cells used than the primary dataset and to achieve stable cell clusters, all other processes were the same as the pipeline described above. The marker genes were calculated and then examined as follows method: If the top marker genes of the cluster were not found in the NET signature, it was defined as a NET negative subtype. If it was found within the NET signature, we defined it as a NET positive subtype marked by the selected genes.

### Generation and evaluation of NET signatures

To investigate the NETosis in bulk sequencing datasets, we generated several sub signatures according to the subtypes of NET positive neutrophils (Supplemental Table 3). These signatures together with the NET signature and T terminal exhaust signature from Zheng et al. ^58^ were input to perform gene set variation analysis (GSVA) by GSVA R package (v1.44.5), evaluating the enrichment score. HALLMARK 50 gene sets downloaded from MSigDB (http://software.broadinstitute.org/gsea/msigdb) were investigated by AUCell R package (v1.20.2). To match the neutrophils defined in this work with the previously reported subtypes^18^, marker genes of tumor associated neutrophils were used to calculate the gene module score with the AddModuleScore function in the Seurat package.

### Construction of gene regulatory network

To generate a gene regulatory network, Single-Cell rEgulatory Network Inference and Clustering (SCENIC) computational method was applied to infer Gene Regulatory Networks (GRN) and cell types from single-cell RNA-seq data^59^. Briefly, SCENIC generates a potential GRN based on the co-expression between TFs and their target genes and then corrects false positive GRN by enrichment score based on an experimental ChIP-Seq based motif database. In this work, single cell RNA sequencing data was processed in Python and pySCENIC (v0.12.0) was applied.

### Cell-Cell communication analysis

Cellchat R package (v1.6.1) was applied to analyze cell-cell communication based on ligands and receptors inferred, implemented with the normalized expression matrix from Seurat with default database and parameters. The netVisual_bubble function was used to visualize the communication interactions between macrophages and neutrophils divided into different subtypes.

### Single-cell pseudotrajectory analysis

Slingshot R package (v2.4.0) was used to determine the single-cell pseudo-time trajectory. The neutrophils expression matrix following NMF reduction was input to Slingshot downstream with default parameters. The origin cell cluster was set as NET negative neutrophils.

### PROGENy analysis

Pathway RespOnsive GENes for activity inference from gene expression analysis was conducted using the progeny R package (v1.18.0). The PROGENy weights were calculated for respective pathway scores. The pathway scores were scaled according to cell types or NET subtypes.

### Live-cell visualization of NETosis and ROS

Neutrophils were isolated from 5 ml whole blood from eight clinically confirmed pancreatic cancer patients using EasySep™ HLA Chimerism Whole Blood CD66b Positive Selection Kit (StemCell) following the manufacturer’s instructions. Neutrophils were plated at 10,000 cells per well (100 μl per well) in serum-free Ham’s F-12K medium (Gibco) using 96-well flat-bottom plates coated with 0.01% poly-l-ornithine solution (Sigma). Then, 250 nM IncuCyte Cytotox Green Dye (Sartorius) and 5uM CellROX Deep Red (Thermo Fisher) were added to measure NETosis and ROS, respectively. IncuCyte Cytotox reagent fluoresces when binding to DNA, allowing for real-time observation of NET release^60^. CellROX Deep Red is a cell-permeant probe that detects intracellular superoxide anions and hydroxyl radicals^61^. After incubation for 2 hours, the detection and quantification of neutrophil fluorescence were taken by a PerkinElmer Opera Phenix Plus high‐content screening system.

### Multi-color immunofluorescence staining

We collected pathologically confirmed pancreatic cancer specimens from 20 patients undergoing radical pancreatic cancer surgery from the Peking Union Medical College Hospital (PUMCH) with the approval of the Institutional Ethics Committee of PUMCH (Ethic code: I-22PJ487). Twenty surgical pancreatic cancer specimens were fixed in formalin and then embedded in paraffin. Multispectral IF staining was performed as previously described^62^. These tumor tissue sections were incubated with the following antibodies: anti-CD66b (Abcam, ab207718, dilute at 1:500), anti-Myeloperoxidase (Abcam, ab208670, dilute at 1:100), anti-CD68 (Abcam, ab213363, dilute at 1:100), and anti-LC3B (Abcam, ab192890, dilute to 1 µg/ml). Nuclear staining was performed with ProLong Diamond Antifade mounting medium containing DAPI (Invitrogen). Images of tissue specimens were obtained using the TissueFAXS Spectra Systems (TissueGnostics GmbH, Vienna Austria). With the help of StrataQuest analysis software (Version 7.1.129, TissueGnostics GmbH, Vienna, Austria), we separated the multi spectral image (5-color staining) and set a threshold to divide the positive cells for analysis of cell number and location distribution.

### Estimation of immune infiltration

The tumor Immune Estimation Resource database (https://cistrome.org/TIMER) investigates the immune infiltrates in various cancer types including PDAC and was applied to calculate the correlation between gene expression and neutrophil or macrophage infiltration in the TCGA-PAAD cohort. The CIBERSORT and TIMER results were used for macrophages and neutrophils. The scatterplot showed the purity-corrected partial Spearman’s correlation and statistical significance that were provided within the datasets.

### Statistical analysis

Statistical analyses were conducted by R software (v4.2.1). Two-tailed unpaired student’s t-test was utilized to show the significant difference between groups. A *p*-value of < 0.05 was considered to indicate a statistically significant result. Significant differences were noted as * *p*-value < 0.05, ** *p*-value < 0.01, and *** *p*-value < 0.001. Results were visualized using the ggplot2 R package (v3.4.2). The Kaplan-Meier survival analysis was performed by comparing the survival data of overall survival time and log-rant test was applied. Survival modeling was undertaken using the survival R package (v3.5-5) and the cutoff value was determined by the survminer R package (v0.4.9).

### Reporting summary

Further information on research design is available in the Nature Research Reporting Summary linked to this article.

### Supplementary information

Supplementary Information

Reporting Summary

## Data Availability

The raw sequence data included in this study was retrieved from the Genome Sequence Archive (GSA, https://ngdc.cncb.ac.cn/) under the accession number GSA:001160 and GEO database (https://www.ncbi.nlm.nih.gov/geo/) under accession number GSE154778, GSE111672 and GSE212966. The TCGA-PAAD dataset was obtained from USCS Xena (https://xena.ucsc.edu/). The ICGC cohort was obtained from the ICGC database ICGC-PACA-AU cohorts.

## Code availability

This study did not generate original codes. All software and algorithms used in this study are publicly available and listed in the Method section. R codes used to analyze data and generate figures are available upon request.
