# Supplementary material for: Multiomics integration reveals NETosis heterogeneity and TLR2 as a prognostic biomarker in pancreatic cancer
Source: NPJ Precis Oncol. 2024 May 20;8:109. doi: 10.1038/s41698-024-00586-x (PMC11106236; doi:10.1038/s41698-024-00586-x)
Supplement: Supplementary file 1 — Supplementary Information [file 41698_2024_586_MOESM1_ESM.pdf]

Supplementary Information

1. **Supplementary Figure 1-4**
2. **Supplementary Table 1-3**

Supplementary Figure 1: **a**: UMAP plot before (left) and after (right) batch effect correction; **b**: Cell counts of included neutrophils in each dataset; **c**: UMAP plot displaying single cells colored by seven distinct clusters; **d**: Clusters of neutrophils at different resolutions (0.1 - 0.9); **e**: The NET score of tumor samples and normal (healthy donors) samples; **f**: Profiling the expression pattern in previous study from Wang *et al.* and this work.

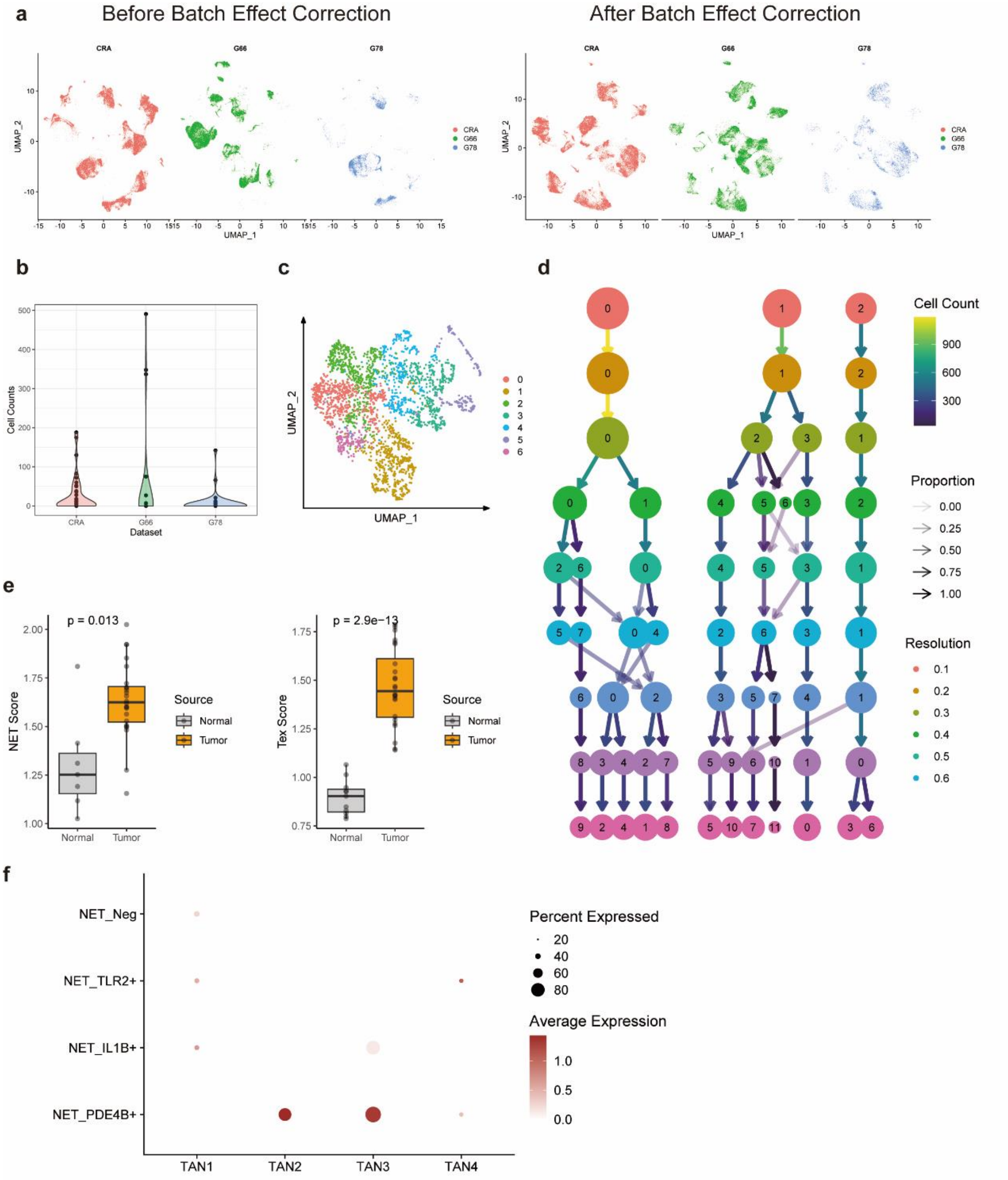

Supplementary Figure 2: **a**: Boxplot shows downregulated ROS score among NET positive neutrophil subtypes; **b**: Boxplot suggested upregulated of inflammatory response and TNF-α via NF-κB pathway in NET positive neutrophil subtypes; **c**: Pathway activities were clustered by NET state; **d**: Pathway activation of Glycolysis, Hypoxia and Oxidative Phosphorylation in different neutrophils subtypes. The scores of NET negative neutrophils were significantly higher than NET positive neutrophils, suggesting a ROS-related metabolic reprogramming.

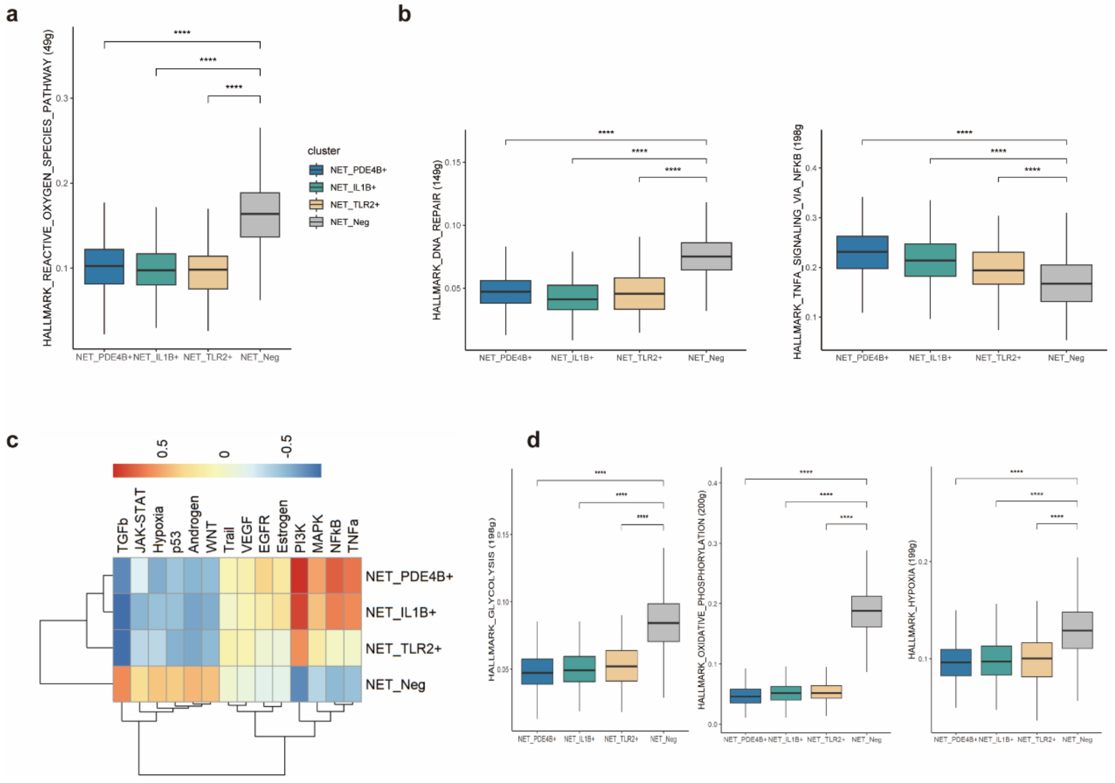

Supplementary Figure 3: **a**: Cell-cell interaction analysis reveals the active interaction between NET_Neg neutrophils and macrophages, while NET positive neutrophils showed inactive interaction; **b**: Boxplot profiles the counts of neutrophils at different distances from macrophages, left: 20um and right: 40um; **c**: The communication signal of outgoing (left) and incoming (right) of macrophages and neutrophils. The color represented the identity of communication; **d**: Dotplot profiled lagend and receptor from Mφ to neutrophils, and the color of the dots showed the connection strength; **e**: The pseudotrajectory of neutrophils in UMAP revealed the potential isogeneity between NET_IL1B+ and NET_PDE4B+; **f**: Correlation of expression pattern between *IL1B* and *PDE4B*.

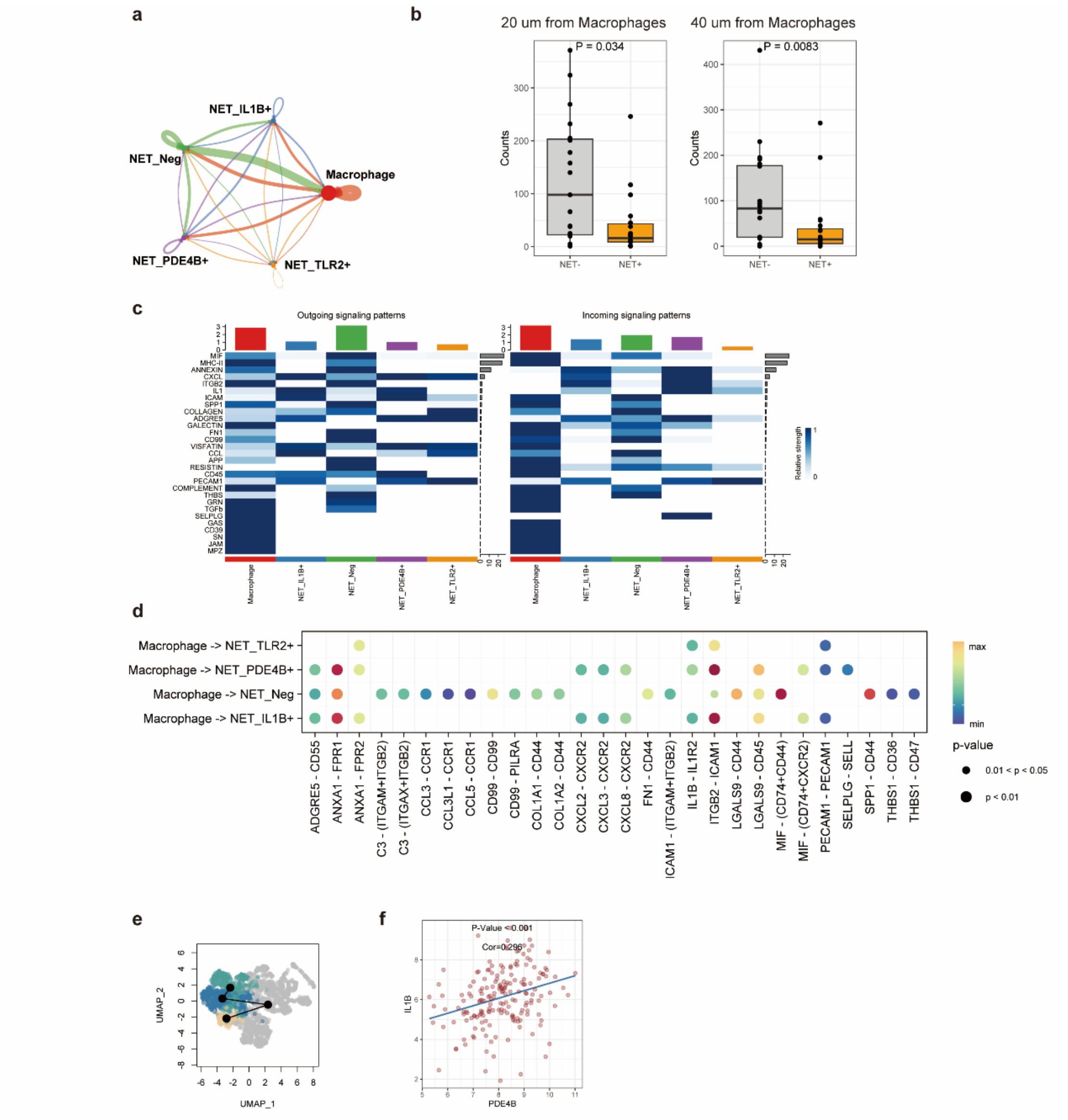

Supplementary Figure 4: **a**: Relative expression of IL1B, PD4EB and TLR2 were shown in UMAP plot. The IL1B and TLR2 were specifically expressed in neutrophil, while the PDE4B had a wide expression; **b**: Immune infiltration analysis; **c**: The prognosis model of PDE4B; **d**: Spatial transcriptome data suggested different location of neutrophils and macrophages.

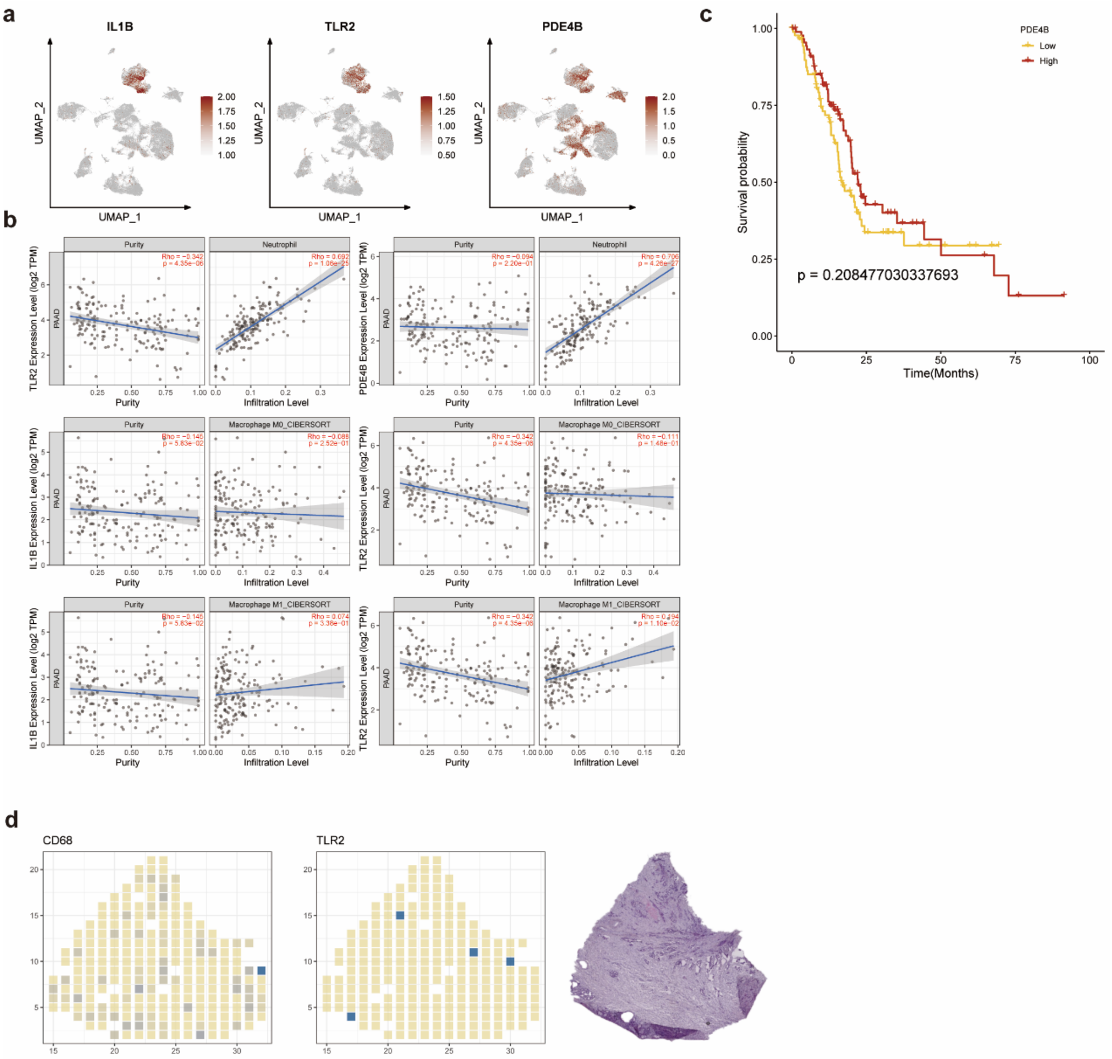

Supplementary Table 1 The list of primer sequences in the analysis

Gene	Forward	Reverse
GAPDH	5′-ACCCACTCCTCCACCTTTGA-3′	5′-CATACCAGGAAATGAGCTTGACAA-3′
TLR2	5'-CTACCAGATGCCTCCCTCTTACCC-3'	5'-TGCCACCAGCTTCCAAAGTCTTC-3'
MS4A6A	5'-CAGAGAAACCCGAACCCACCAAC-3'	5'-GCAGATGCCAAAATGATCCCCAAG-3'
HAVCR2	5'-AATACAGAGCGGAGGTCGGTCAG-3'	5'-CGTTGCCACATTCAAACACAGGAC-3'
CD27	5'-GCCCTGTTCCTCCATCAACGAAG-3'	5'-GATGGTGCTGCCCTCCTCCTC-3'
RGS1	5'-TTGAGTTCTGGCTGGCTTGTGAAG-3'	5'-GCTGTAGATTCTCGAGTGCGGAAG-3'
SIRPG	5'-GGCAGGTGAGGAGGAGCTACAG-3'	5'-CCACGGGAAGCAGGGAGGTC-3'
CTLA4	5'-CTGAAGTCTGTGCGGCAACCTAC-3'	5'-TGGCCCTCAGTCCTTGGATAGTG-3'
TIGIT	5'-TGCCAGGTTCCAGATTCCATTGC-3'	5'-GCGACCACCACGATGACTGC-3'
HMOX1	5'-TGCCAGTGCCACCAAGTTCAAG-3'	5'-TGTTGAGCAGGAACGCAGTCTTG-3'
LAG3	5'-CTGGAGACAATGGCGACTTTACCC-3'	5'-GCAGATGGATATGGCAGGTGTAGG-3'
PDCD1	5'-GCCGTGCCTGTGTTCTCTGTG-3'	5'-TGAGGTGCCCATTCCGCTAGG-3'

Supplementary Table 2 NET signatures

Functional Pathway	NET Signature
Neutrophil activation	ALPL
Neutrophil activation	BST1
Neutrophil activation	CD93
Neutrophil activation	CEACAM3
Neutrophil activation	CREB5
Neutrophil activation	CRISPLD2
Neutrophil activation	CSF3R
Neutrophil activation	CYP4F3
Neutrophil activation	DYSF
Neutrophil activation	FCAR
Neutrophil activation	FCGR3B
Neutrophil activation	CPPED1
Neutrophil activation	FPR1
Neutrophil activation	FPR2
Neutrophil activation	G0S2
Neutrophil activation	HIST1H2BC
Neutrophil activation	HPSE
Neutrophil activation	CXCR1
Neutrophil activation	CXCR2
Neutrophil activation	KCNJ15
Neutrophil activation	LILRB2
Neutrophil activation	MGAM
Neutrophil activation	MME
Neutrophil activation	PDE4B
Neutrophil activation	S100A12
Neutrophil activation	SIGLEC5
Neutrophil activation	SLC22A4
Neutrophil activation	SLC25A37
Neutrophil activation	TECPR2
Neutrophil activation	TNFRSF10C
Neutrophil activation	VNN3
NETosis	AKT1
NETosis	AKT2
NETosis	ATG7
NETosis	CLEC6A
NETosis	CSF3
NETosis	CTSG
NETosis	CYBB
NETosis	DNASE1
NETosis	ELANE
NETosis	ENTPD4
NETosis	F3
NETosis	HMGB1
NETosis	IL17A
NETosis	IL1B
NETosis	IL6
NETosis	IL8
NETosis	IRAK4

NETosis	ITGAM
NETosis	ITGB2
NETosis	KCNN3
NETosis	MAPK1
NETosis	MAPK3
NETosis	MMP9
NETosis	MPO
NETosis	MTOR
NETosis	PADI4
NETosis	PTAFR
NETosis	PIK3CA
NETosis	RIPK1
NETosis	RIPK3
NETosis	SELP
NETosis	SELPLG
NETosis	SIGLEC14
NETosis	TLR2
NETosis	TLR4
NETosis	TLR7
NETosis	TLR8
NETosis	TNF

Supplementary Table 3 NET subtype signatures

NET_subtype	marker
NET_PDE4B+	PDE4B
NET_PDE4B+	NAMPT
NET_PDE4B+	IFITM2
NET_PDE4B+	G0S2
NET_PDE4B+	FCGR3B
NET_PDE4B+	SRGN
NET_PDE4B+	LITAF
NET_PDE4B+	FTH1
NET_PDE4B+	PLAUR
NET_PDE4B+	CSF3R
NET_PDE4B+	SOD2
NET_PDE4B+	BCL2A1
NET_PDE4B+	CXCL8
NET_PDE4B+	IVNS1ABP
NET_PDE4B+	RNF149
NET_PDE4B+	IL1B
NET_PDE4B+	HLA-B
NET_PDE4B+	LUCAT1
NET_PDE4B+	TNFAIP3
NET_PDE4B+	BTG2
NET_PDE4B+	ITM2B
NET_PDE4B+	NEAT1
NET_PDE4B+	CMTM2
NET_PDE4B+	TREM1
NET_PDE4B+	B2M
NET_PDE4B+	MXD1
NET_PDE4B+	H3F3B
NET_PDE4B+	MCL1
NET_PDE4B+	CCL4L2
NET_PDE4B+	LCP1
NET_PDE4B+	LCP2
NET_PDE4B+	HLA-C
NET_PDE4B+	SAT1
NET_PDE4B+	SLC25A37
NET_PDE4B+	HLA-E
NET_PDE4B+	SMCHD1
NET_PDE4B+	S100A9
NET_PDE4B+	IER3
NET_PDE4B+	PTGS2
NET_PDE4B+	H3F3A
NET_PDE4B+	ICAM1
NET_PDE4B+	ALOX5AP
NET_PDE4B+	PLEK
NET_PDE4B+	CXCR2
NET_PDE4B+	ADGRG3
NET_PDE4B+	FPR1
NET_PDE4B+	OSM
NET_PDE4B+	IL1R2

NET_PDE4B+	LAPTM5
NET_PDE4B+	CEBPB
NET_PDE4B+	RGS2
NET_PDE4B+	MALAT1
NET_PDE4B+	TMEM154
NET_PDE4B+	C5AR1
NET_PDE4B+	ELL2
NET_PDE4B+	PTPRC
NET_PDE4B+	SAMSN1
NET_PDE4B+	PPIF
NET_PDE4B+	NFKBIA
NET_PDE4B+	MARCKS
NET_PDE4B+	ACTB
NET_PDE4B+	LST1
NET_PDE4B+	GNG10
NET_PDE4B+	NIBAN1
NET_PDE4B+	DUSP1
NET_PDE4B+	TRIB1
NET_PDE4B+	N4BP1
NET_PDE4B+	PNRC1
NET_PDE4B+	GPR65
NET_PDE4B+	PHACTR1
NET_PDE4B+	EGR3
NET_PDE4B+	SMAP2
NET_PDE4B+	SDCBP
NET_PDE4B+	PPP1R15A
NET_PDE4B+	TNFAIP6
NET_PDE4B+	KDM6B
NET_PDE4B+	CSRNP1
NET_PDE4B+	MNDA
NET_PDE4B+	CXCR4
NET_PDE4B+	SYAP1
NET_PDE4B+	EHD1
NET_PDE4B+	CCL4
NET_PDE4B+	SERPINA1
NET_PDE4B+	MX2
NET_PDE4B+	RIPOR2
NET_PDE4B+	GLUL
NET_PDE4B+	S100A8
NET_PDE4B+	CD55
NET_PDE4B+	AQP9
NET_PDE4B+	CREM
NET_PDE4B+	UBE2B
NET_PDE4B+	IFRD1
NET_PDE4B+	NABP1
NET_PDE4B+	SEC14L1
NET_PDE4B+	PHF20L1
NET_PDE4B+	BASP1
NET_PDE4B+	CCL3L1
NET_PDE4B+	NFE2L2

NET_PDE4B+	TPM4
NET_PDE4B+	ISG20
NET_PDE4B+	SKIL
NET_PDE4B+	SLA
NET_PDE4B+	VPS37B
NET_PDE4B+	YPEL5
NET_PDE4B+	SORL1
NET_PDE4B+	ABHD5
NET_PDE4B+	RILPL2
NET_PDE4B+	VASP
NET_PDE4B+	CPD
NET_PDE4B+	CHD1
NET_PDE4B+	HIF1A
NET_PDE4B+	ANP32A
NET_PDE4B+	IRS2
NET_PDE4B+	ACSL1
NET_PDE4B+	FOXO3
NET_PDE4B+	GNAI3
NET_PDE4B+	TXNIP
NET_PDE4B+	ACTG1
NET_PDE4B+	NFKBIZ
NET_PDE4B+	MAP3K8
NET_PDE4B+	NR4A3
NET_PDE4B+	ETS2
NET_PDE4B+	IRAK3
NET_PDE4B+	PROK2
NET_PDE4B+	IL1RN
NET_PDE4B+	ISG15
NET_PDE4B+	IFIT2
NET_PDE4B+	CXCL1
NET_PDE4B+	RSAD2
NET_PDE4B+	IFIT3
NET_IL1B+	CCL4L2
NET_IL1B+	CCL3L1
NET_IL1B+	IL1B
NET_IL1B+	G0S2
NET_IL1B+	TNFAIP3
NET_IL1B+	FTH1
NET_IL1B+	CCL4
NET_IL1B+	CXCL8
NET_IL1B+	IER3
NET_IL1B+	NAMPT
NET_IL1B+	SOD2
NET_IL1B+	BCL2A1
NET_IL1B+	PHACTR1
NET_IL1B+	IFITM2
NET_IL1B+	FCGR3B
NET_IL1B+	ITM2B
NET_IL1B+	ALOX5AP
NET_IL1B+	PLAUR

NET_IL1B+	MXD1
NET_IL1B+	PNRC1
NET_IL1B+	NFKBIA
NET_IL1B+	CXCR4
NET_IL1B+	RNASEK
NET_IL1B+	LUCAT1
NET_IL1B+	CSF3R
NET_IL1B+	NEAT1
NET_IL1B+	LITAF
NET_IL1B+	AZIN1-AS1
NET_IL1B+	HLA-E
NET_IL1B+	BTG2
NET_IL1B+	IVNS1ABP
NET_IL1B+	CCL3
NET_IL1B+	PPP1R15A
NET_IL1B+	ICAM1
NET_IL1B+	SLC25A37
NET_IL1B+	MALAT1
NET_IL1B+	CEBPB
NET_IL1B+	PPIF
NET_IL1B+	MCL1
NET_IL1B+	TNFAIP6
NET_IL1B+	LAPTM5
NET_IL1B+	MARCKS
NET_IL1B+	MT1X
NET_IL1B+	TREM1
NET_IL1B+	ATP6V0C
NET_IL1B+	PLEK
NET_IL1B+	FPR1
NET_IL1B+	C15orf48
NET_IL1B+	SRGN
NET_IL1B+	LST1
NET_IL1B+	LCP1
NET_IL1B+	CREM
NET_IL1B+	H3F3B
NET_IL1B+	OSM
NET_IL1B+	C5AR1
NET_IL1B+	KDM6B
NET_IL1B+	RGS2
NET_IL1B+	MT2A
NET_IL1B+	LCP2
NET_IL1B+	DUSP1
NET_IL1B+	JUNB
NET_IL1B+	SAMSN1
NET_IL1B+	TXNIP
NET_IL1B+	CD83
NET_IL1B+	IL1A
NET_IL1B+	NFE2L2
NET_IL1B+	ELL2
NET_IL1B+	PTGS2

NET_IL1B+	TSC22D3
NET_IL1B+	FOSB
NET_IL1B+	TAGAP
NET_IL1B+	IL1RN
NET_IL1B+	EGR1
NET_IL1B+	NFKBIZ
NET_IL1B+	FOS
NET_TLR2+	TLR2
NET_TLR2+	CXCR4
NET_TLR2+	PHACTR1
NET_TLR2+	CCL3L1
NET_TLR2+	NEAT1
NET_TLR2+	MALAT1
NET_TLR2+	TXNIP
NET_TLR2+	FCGR3B
NET_TLR2+	ATP6V0C
NET_TLR2+	FTH1
NET_TLR2+	NAMPT
NET_TLR2+	B2M
NET_TLR2+	G0S2
NET_TLR2+	ALOX5AP
NET_TLR2+	LITAF
NET_TLR2+	RNASEK
NET_TLR2+	TMBIM4
NET_TLR2+	SEC14L1
NET_TLR2+	MXD1
NET_TLR2+	RESF1
NET_TLR2+	IFITM2
NET_TLR2+	HLA-E
NET_TLR2+	ITM2B
NET_TLR2+	CREM
NET_TLR2+	CXCL8
NET_TLR2+	TSC22D3
NET_TLR2+	CSF3R
NET_TLR2+	IER3
NET_TLR2+	LUCAT1
NET_TLR2+	UBC
NET_TLR2+	SPAG9
NET_TLR2+	MAP1LC3B
NET_TLR2+	CEBPB
NET_TLR2+	IRS2
NET_TLR2+	GABARAP
NET_TLR2+	AL499604.1
NET_TLR2+	ASAH1
NET_TLR2+	SMIM25
NET_TLR2+	IVNS1ABP
NET_TLR2+	H3F3B
NET_TLR2+	BRI3
NET_TLR2+	C15orf48
NET_TLR2+	MARCKS

NET_TLR2+	KATNBL1
NET_TLR2+	ABHD5
NET_TLR2+	UBE2B
NET_TLR2+	MAFF
NET_TLR2+	CCL3
NET_TLR2+	IL1R2
NET_TLR2+	IGKC
NET_TLR2+	DUSP1
NET_TLR2+	TANK
NET_TLR2+	DDIT3
NET_TLR2+	C5AR1
NET_TLR2+	ANP32A
NET_TLR2+	CPD
NET_TLR2+	FOS
NET_TLR2+	IFI30
